# Enhancement of ethanol production from green liquor–ethanol-pretreated sugarcane bagasse by glucose–xylose cofermentation at high solid loadings with mixed *Saccharomyces cerevisiae* strains

**DOI:** 10.1186/s13068-017-0771-7

**Published:** 2017-04-13

**Authors:** Yanzhi You, Pengfei Li, Fuhou Lei, Yang Xing, Jianxin Jiang

**Affiliations:** 1 Department of Chemistry and Chemical Engineering, MOE Engineering Research Center of Forestry Biomass Materials and Bioenergy, Beijing Forestry University, Beijing, 100083 China; 2 GuangXi Key Laboratory of Chemistry and Engineering of Forest Products, College of Chemistry and Chemical Engineering, Guangxi University for Nationalities, Nanning, 530006 China

**Keywords:** Ethanol, Sugarcane bagasse, GL–Ethanol pretreatment, Glucose–xylose cofermentation, *Saccharomyces cerevisiae*

## Abstract

**Background:**

Efficient cofermentation of glucose and xylose is necessary for economically feasible bioethanol production from lignocellulosic biomass. Here, we demonstrate pretreatment of sugarcane bagasse (SCB) with green liquor (GL) combined with ethanol (GL–Ethanol) by adding different GL amounts. The common *Saccharomyces cerevisiae* (CSC) and thermophilic *S. cerevisiae* (TSC) strains were used and different yeast cell mass ratios (CSC to TSC) were compared. The simultaneous saccharification and cofermentation (SSF/SSCF) process was performed by 5–20% (w/v) dry substrate (DS) solid loadings to determine optimal conditions for the co-consumption of glucose and xylose.

**Results:**

Compared to previous studies that tested fermentation of glucose using only the CSC, we obtained higher ethanol yield and concentration (92.80% and 23.22 g/L) with 1.5 mL GL/g-DS GL–Ethanol-pretreated SCB at 5% (w/v) solid loading and a CSC-to-TSC yeast cell mass ratio of 1:2 (w/w). Using 10% (w/v) solid loading under the same conditions, the ethanol concentration increased to 42.53 g/L but the ethanol yield decreased to 84.99%. In addition, an increase in the solid loading up to a certain point led to an increase in the ethanol concentration from 1.5 mL GL/g-DS-pretreated SCB. The highest ethanol concentration (68.24 g/L) was obtained with 15% (w/v) solid loading, using a CSC-to-TSC yeast cell mass ratio of 1:3 (w/w).

**Conclusions:**

GL–Ethanol pretreatment is a promising pretreatment method for improving both glucan and xylan conversion efficiencies of SCB. There was a competitive relationship between the two yeast strains, and the glucose and xylose utilization ability of the TSC was better than that of the CSC. Ethanol concentration was obviously increased at high solid loading, but the yield decreased as a result of an increase in the viscosity and inhibitor levels in the fermentation system. Finally, the SSCF of GL–Ethanol-pretreated SCB with mixed *S. cerevisiae* strains increased ethanol concentration and was an effective conversion process for ethanol production at high solid loading.

## Background

Biomass residues from forestry, agriculture, or dedicated perennial (energy) crops are potential feedstocks for fermentative ethanol production, which can minimize greenhouse gas emissions from the use of petroleum-based transportation fuels [1] and provide a renewable energy source to combat the diminishing global fossil fuel supply [2]. During ethanol production from such lignocellulosic materials, it is important to use all sugars available, i.e., both hexoses and pentoses, to obtain a high yield [3].

The yeast *Saccharomyces cerevisiae* (*S. cerevisiae*) can rapidly ferment hexoses, especially glucose, but is unable to metabolize xylose naturally. For example, the hydrolysis of hemicellulose generates substantial amounts of pentose sugars that cannot be fermented by wild-type *S. cerevisiae*. Additionally, plant hydrolysates contain substances inhibitory from the biomass or pretreatment applied, which may inhibit microbial growth during the fermentation process [4]. Therefore, several technological advancements are required, including the development of cost-effective cellulosic biomass pretreatment and hydrolysis processes [5] and the engineering of robust industrial microbes that are capable of fermenting mixed streams of hexoses and pentoses derived from lignocellulosic biomass [6]. Many attempts have been made to introduce and optimize heterologous metabolic pathways for xylose utilization in *S. cerevisiae*, such as the oxidoreductase-based pathway with xylose reductase and xylitol dehydrogenase and the isomerase-based pathway with xylose isomerase [7, 8]. Considering the cofactor imbalance associated with the oxidoreductase pathway, the development of *S. cerevisiae* capable of xylose utilization via a xylose isomerase-based pathway has been considered as the most promising strategy [9, 10]. Ho et al. [11] made a breakthrough by creating super-stable genetically engineered glucose–xylose-cofermenting *Saccharomyces* yeasts that contain multiple copies of the same three xylose-metabolizing genes stably integrated in the yeast chromosome, which made it possible to move the biomass-to-ethanol technology by the continuous cofermentation of glucose and xylose much closer to commercialization. Therefore, one significant challenge is to achieve efficient and simultaneous uptake of pentose and hexose sugars in the fermentation process [12]. Another limitation of cellulosic ethanol production is the difficulty of using a high solid loading in simultaneous saccharification and cofermentation (SSF/SSCF), which restricts the final ethanol concentration [13].

Ethanol production from lignocellulosic biomass comprises the following main steps: hydrolysis of cellulose and hemicellulose, sugar fermentation, separation of lignin residue, and finally recovery and purification of ethanol to meet the fuel specifications. The task of hydrolyzing lignocellulose to fermentable monosaccharides is still technically problematic because the digestibility of cellulose is hindered by many physico-chemical, structural, and compositional factors [5]. Pretreatment eliminates the physical and chemical barriers that make native biomass recalcitrant and allows cellulose to become amenable to enzymatic hydrolysis, which is a critical step in the biochemical processing of lignocellulose, based on the “sugar platform” concept. This effect is achieved by increasing the accessible cellulose surface area through the solubilization of hemicelluloses and/or lignin, which coat the cellulose of the native biomass [14]. Various pretreatment approaches have been investigated with extensive feedstocks, focusing on the enzymatic hydrolysis of the treated biomass, and use of lower enzyme dosages and shorter bioconversion times. Several recent review articles provide a general overview of this field [15–18].

Since different lignocellulosic materials have different physico-chemical characteristics, it is necessary to adopt appropriate pretreatment technologies based on their biomass properties. Furthermore, the choice of pretreatment has a large impact on all the subsequent steps in the overall conversion scheme in terms of cellulose digestibility, generation of toxic compounds that potentially inhibit yeast growth, stirring power requirements, energy demand in the downstream process, and wastewater treatment demands [19]. Several physical–chemical pretreatment methods have been investigated for these purposes, including steam and ammonia fiber explosion; hydrothermal methods; peroxidation; acid hydrolysis with concentrated or diluted sulfuric, hydrochloric, phosphoric or peracetic acid; alkaline hydrolysis; the organosolv process; and irradiation using ionizing rays, ultrasonic waves, and microwaves [20]. Among the pretreatment methods, alkaline and organosolv pretreatments have been widely employed for improving the yield of fermentable sugars following enzymatic hydrolysis [21].

Green liquor (GL), an alkaline liquid, is produced from the pulping process and its composition varies with the pulping methods used. The GL produced from the soda pulping process is a mixture of sodium carbonate and sodium hydroxide. Since it causes little environmental contamination, the soda pulping method has become a significant alternative in some mills in China [21]. The use of GL in an alkaline pretreatment process has been recently developed to improve bio-fuel production [22]. The method using moderate alkaline conditions tends to selectively remove lignin and leave both the hemicelluloses and cellulose fractions in the pulp for subsequent conversion to fermentable sugars. Currently, the use of GL from soda pulping mills has been developed as a pretreatment method to improve fermentable sugar generation, as it is environmentally friendly [23, 24]. In addition, organosolv pretreatment is milder than organosolv pulping, and has some typical advantages compared with those of other pretreatments: for example, (1) the lignin degradation products can be applied in the fields of adhesives, films, biodegradable polymers, and other coproducts; and (2) the organic solvents can be easily recovered and recycled by distillation. Among these organosolv pretreatments, ethanol pretreatment is the preferred method because of the low toxicity and low boiling point of ethanol, making it easily recycled by distillation. Reports have shown that GL combined with ethanol (GL–Ethanol) is a promising pretreatment method for improving both glucan and xylan conversion efficiencies. Besides, the reactive lignin removed from GL to Ethanol pretreatment can be applied in the fields of biodegradable polymer, adhesive, and other value-added products [24–26].

Enzymatic hydrolysis can be conducted simultaneously with the cofermentation of glucose and xylose, and is referred to as SSCF. Compared with the separate hydrolysis and fermentation (SHF) and the separate hydrolysis and cofermentation (SHCF) methods, SSCF offers several advantages, including continuous removal of hydrolysis end-products that inhibit enzymes, and low contamination risk. SSCF is also superior to SSF owing to the high productivity and yield of ethanol [13, 27]. However, in addition to yield, the ethanol concentration is also an important factor, as the distillation costs decrease as a function of the final ethanol concentration [28]. To increase the ethanol concentration, a high loading of solid is needed. An ethanol concentration higher than 4% (w/v) in the fermentation broth is the benchmark for efficient distillation, considering the energy consumption and efficiency of the ethanol recovery process [29, 30]. For this, it is recommended that the hydrolysis and fermentation processes should be conducted at an initial solid loading higher than 10% (w/v) for agricultural straw. However, with further increases in solid loading, the ethanol yield decreases as a result of increased mass transfer resistance, inhibitory effects, and decreased xylose uptake in the SSCF [31, 32].

In this study, GL–Ethanol was chosen for treating sugarcane bagasse (SCB). The strains of common *S. cerevisiae* (CSC) and thermophilic *S. cerevisiae* (TSC) with different yeast cell mass ratios (CSC to TSC) of 1:3, 1:2, 1:1, 2:1, and 3:0 (w/w) were compared at 5–20% (w/v) DS solid loadings to determine the suitable conditions for the co-consumption of glucose and xylose in the SSF/SSCF process. Based on pre-experiments with the two yeast strains using glucose alone, xylose alone, and a mixture of glucose and xylose (data not shown), we finally determined that the optimum fermentation temperature is 35 °C in the current study. Fermentation parameters that might affect the cofermentation performance of glucose and xylose (including pretreatment conditions, CSC-to-TSC yeast cell mass ratios, and solid loadings) were also compared.

## Methods

### Raw material

Raw SCB, composed of 46.97% glucan, 22.44% xylan, 19.48% Klason lignin, and 1.53% ash, was kindly provided by Guitang Corporation (Guangxi, China). It was air-dried and placed at room temperature in plastic bags. The raw SCB was ground and screened with 40 meshes. Those through the 40 meshes were collected as the experimental samples. The GL was supplied by Chenming Group (Shandong, China), and purified by filter paper prior to use in the pretreatment of SCB. 1 mL GL is equal to 1.1660 g GL. The main components of GL were sodium carbonate (75.2 ± 0.25 g/L) and sodium hydroxide (23.04 ± 0.25 g/L). There were also other metal elements in GL, such as iron (1.14 ± 0.08 g/L) and calcium (0.39 ± 0.03 g/L) [26]. All the chemicals used in this study were of analytical grade. The polytetrafluoroethylene (PTFE) reaction vessels were assembled in a commercial device (GS-L reactor) that was purchased from Weihai Jiayi Chemical Machinery Co., Ltd (Shandong, China), and specially used for pretreatments.

### Pretreatment of sugarcane bagasse by GL–Ethanol

GL–Ethanol pretreatment was carried out in a PTFE reactor with a total volume of 200 mL according to the previous study [26]. 10 g raw SCB was pretreated by 0.8, 1.0, and 1.5 mL GL/g-DS at 140 °C for 3 h with a solid/liquid ratio (w/v) of 1:10, respectively. The liquid was a 50:50% (v/v) ethanol: water mixture, which was the optimized condition from our group [26]. Anthraquinone with 1% (w/w, DS) (AQ, Sigma Co., St. Louis, MO, USA) was used for avoiding excessive carbohydrate degradation in GL–Ethanol pretreatment. The system (PTFE reactor + stainless steel tank) was placed in a chamber equipped with a shaft where the PTFE reactor was fixed well with a large stainless steel tank. The system was heated at an average rate of 5 °C/min and rotated at 100 rpm to reach a desired temperature of 140 °C. The system was rapidly cooled with tap water after pretreatment. The pretreated SCB was obtained by filtration prior to washing with 200 mL ethanol–water mixture (50:50%, v/v). Then, solid fraction thus obtained was washed with distilled water until neutral pH.

### Microorganisms and enzyme preparation

The common *S. cerevisiae* (CSC) was purchased from Angel Yeast Company (YiChang, China). The thermophilic *S. cerevisiae* (TSC) was kindly provided by Microbiology Department of Beijing Forestry University. Among them, the CSC only utilized glucose, and TSC could mainly exploit glucose as well as next xylose. 3% (w/v) dry yeast with different CSC-to-TSC yeast cell mass ratios of 1:3, 1:2, 1:1, 2:1, and 3:0 (w/w) was activated in a 2% (w/v) glucose solution at 35 °C for 1 h before SSF/SSCF, respectively. Cellulolytic enzymes were Cellic Ctec2 with a cellulase activity of 130 FPU/mL and Novozym 188 with a β-glucosidase activity of 48 IU/mL, respectively, which were both kindly donated by Novozymes A/S (Bagsvaerd, Denmark).

### Simultaneous saccharification and cofermentation (SSF/SSCF)

The SSF/SSCF experiments were performed under nonsterile conditions. A 100-mL conical flask with a special sealing means containing sterile glycerol for the discharge of carbon dioxide, which could reduce the loss of ethanol, was used. The working volume was 60 g. The amount of the enzymes was 20 FPU/g-cellulose for Cellic Ctec2 and 25 IU/g-cellulose for Novozym 188, respectively [33]. The initial inoculum concentration of yeast was about 5 g/L. Organic medium contained yeast extract, 1 g/L; (NH_4_)_2_HPO_4_, 0.5 g/L; MgSO_4_ **·**7H_2_O, 0.025 g/L. pH value of each fermentation sample was adjusted to 5.5 with 10% (w/v) NaOH or HCl solution. In each experiment, SCB in conical flask and nutrients were separately sterilized (121 °C, 20 min). The enzymes, organic medium, and yeast were then added to the conical flask directly. SSF/SSCF of untreated and pretreated SCB were conducted at the solid loadings of 5, 10, 15, and 20% (w/v) DS with different CSC-to-TSC yeast cell mass ratios of 1:3, 1:2, 1:1, 2:1, and 3:0 (w/w) in an air-bath shaker at a speed of 150 rpm and 35 °C in a initial pH of 5.5. Fermentation with raw SCB was as control case [34].

### Analytical methods

The glucan and xylan contents of samples before and after pretreatment were analyzed according to the National Renewable Energy Laboratory (NREL) methods [35]. Acid-insoluble lignin was determined by the TAPPI method (TAPPI T222 om-06 2006). A muffle furnace was used at 550 °C for 4–5 h to calculate the percentage of total ash according to the residue weight.

**Fig. 1:**
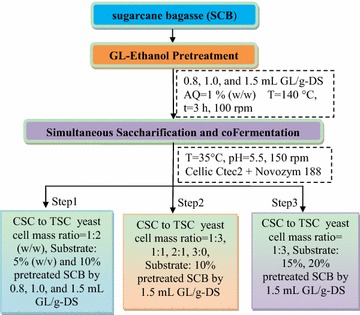
Schematic diagram of experiments

The fermentation samples were filtered (0.22-μm pore) to detect ethanol, glycerol, and acetic acid by high-performance liquid chromatography (HPLC) (Waters e2695, USA) using an Aminex HPX-87H column (300 × 7.8 mm; Bio-Rad Laboratories, USA) at 65 °C with a refractive index detector at 35 °C, and 5 mM sulfuric acid was used as the eluent at a flow rate of 0.6 mL/min. Besides, concentrations of glucose and xylose in the soluble fraction were determined by HPLC using an Aminex HPX-87P (300 × 7.8 mm; Bio-Rad Laboratories, USA) at 85 °C with a refractive index detector at 35 °C, and water (0.6 mL/min) was used as the eluent. The injection volume of the sample was 10 μL, and the total analysis time was 50 min [36, 37]. Assays were performed in two triplicate experiments, and data were presented as the mean of the triplicates. The coefficient of glucose converted from glucan was 1.11 [38]. The coefficient of xylose converted from xylan was 1.14; Based on the maximum theoretical yield of ethanol, ethanol yield was calculated from consumed glucose and xylose, which was 0.51 g ethanol/g glucose and 0.46 g ethanol/g xylose, respectively [31, 34]. The schematic diagram of experiments is illustrated in Fig. 1.

## Results and discussion

### Comparison of the effects of pretreatment methods on the SSF/SSCF process

Table 1 shows the chemical composition of SCB before and after GL–Ethanol pretreatment. For both the untreated and pretreated SCB, the carbohydrates (glucan and xylan) accounted for more than 65% of the DS. These values are significant for the biorefinery process, since raw materials with a high glucan and xylan content are preferable substrates for use in bioethanol production [39]. As shown in Table 1, ~64.97% of lignin was removed when 0.8 mL GL/g-DS was used during the GL–Ethanol pretreatment. Moreover, lignin removal increased with higher GL dosage. The highest lignin removal of 80.92% was obtained with the GL loading of 1.5 mL/g-DS, which is consistent with the previously reported results [21].

**Table 1:** Chemical composition of SCB before and after GL–Ethanol pretreatment

Green liquor (mL/g-DS)	Composition (%)				Solid yield (%)
	Glucan	Xylan	Klason lignin	Ash	
Untreated	46.97 ± 0.17	22.44 ± 0.19	19.48 ± 0.13	1.53 ± 0.13	100
0.8	58.52 ± 0.21	26.77 ± 0.16	8.64 ± 0.42	1.11 ± 0.41	78.97 ± 0.30
1.0	61.74 ± 0.15	28.58 ± 0.01	6.77 ± 1.13	1.59 ± 0.20	75.50 ± 0.40
1.5	61.80 ± 0.49	28.59 ± 0.19	5.03 ± 0.07	1.25 ± 0.19	73.90 ± 0.30

All values are based on the oven-dried weight of samples

*SCB* sugarcane bagasse, *GL Ethanol* GL combined with ethanol, *DS* dry substrate

**Fig. 2:**
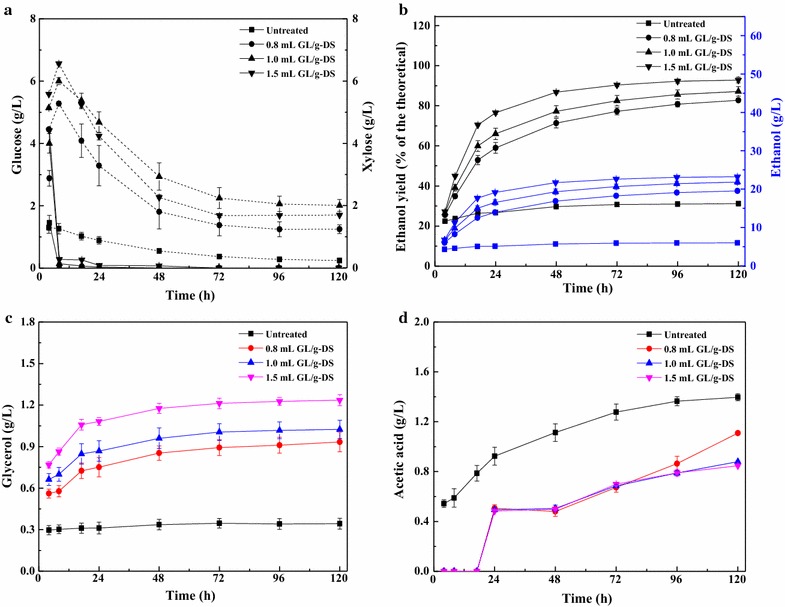
Ethanol fermentation of pretreated sugarcane bagasse (SCB) using 0.8, 1.0, and 1.5 mL GL/g-DS in GL–Ethanol pretreatment at 5% (w/v) solid loading, and a yeast cell mass ratio of 1:2 (w/w) common *S. cerevisiae* (CSC) to thermophilic *S. cerevisiae* (TSC). Fermentation conditions: 150 rpm, 35 °C, pH 5.5, and 120 h. Square, circular, upright triangle and inverted triangle symbols represent untreated, 0.8 mL GL/g-DS, 1.0 mL GL/g-DS and 1.5 mL GL/g-DS pretreated SCB, respectively. **a** Sugars (glucose and xylose) concentration; **b** Ethanol yield (% of the theoretical) and ethanol concentration; **c** Glycerol concentration; and **d** Acetic acid concentration

The concentration profiles of ethanol, glucose, xylose, and the fermentation byproducts, glycerol and acetic acid, are shown for the untreated SCB and for SCB pretreated with 0.8, 1.0, and 1.5 mL GL/g-DS at a 5% (w/v) solid loading and a CSC-to-TSC yeast cell mass ratio of 1:2 (w/w) during the SSCF (Fig. 2). Glucose was rapidly consumed within 8 h, while xylose was utilized only by the TSC after 8 h and achieved an approximately steady state (2–3 g/L unutilized) after 72 h (Fig. 2a). These results imply that glucose was initially consumed by both the CSC and TSC simultaneously, while the TSC probably began to consume xylose once the glucose concentration became low and could thus coferment glucose and xylose effectively [31]. Previous studies have confirmed that glucose concentration is a critical factor in determining cell viability and xylose consumption [3, 27].

Similar profiles of ethanol concentration and yield from the various pretreated SCB samples were observed throughout the SSCF process. The concentration or yield of the fermentation products in the pretreated SCB increased with the increase in GL dosage, compared to their levels in the untreated SCB. The ethanol yield reached 59% of the theoretical maximum within 24 h with 0.8 mL GL/g-DS, and the highest ethanol yield and concentration of 92.80% and 23.22 g/L, respectively, were obtained with 1.5 mL GL/g-DS at 120 h in the SSCF process (Fig. 2b). This is consistent with a previous study where 1.5 mL GL/g-DS was used during GL–Ethanol pretreatment and the highest glucose and xylose yields were obtained after 72 h of enzymatic hydrolysis [21]. However, we obtained lower ethanol yield and concentration from the untreated SCB, probably due to the inhibitory effect of the high content of lignin. These results indicate that GL–Ethanol pretreatment significantly improves the conversion of glucan and xylan because of the removal of large amounts of lignin. Therefore, GL–Ethanol pretreatment is a promising pretreatment method for improving both the glucan and xylan conversion efficiencies of SCB, thus improving the subsequent fermentation efficiency [21]. In this study, we used both the CSC and TSC yeast strains at a cell mass ratio of 1:2 (w/w). In contrast, when only the CSC was used in the SSF process, a low ethanol yield of 80.56% (12.90 g/L) of the similarly pretreated SCB (1.5 mL GL/g-DS) was obtained [24].

**Fig. 3:**
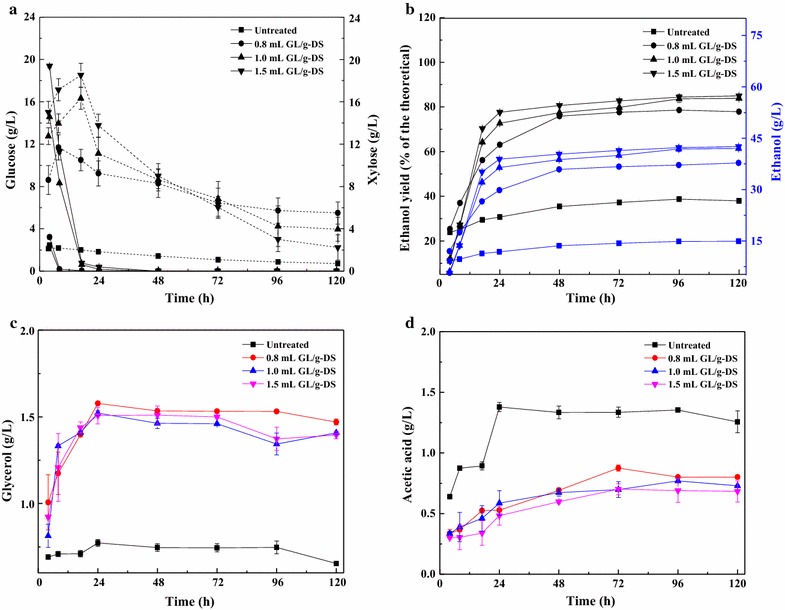
Ethanol fermentation of pretreated sugarcane bagasse (SCB) using 0.8, 1.0, and 1.5 mL GL/g-DS in GL–Ethanol pretreatment at 10% (w/v) solid loading, and a yeast cell mass ratio of 1:2 (w/w) common *S. cerevisiae* (CSC) to thermophilic *S. cerevisiae* (TSC). Fermentation conditions: 150 rpm, 35 °C, pH 5.5, and 120 h. Square, circular, upright triangle and inverted triangle symbols represent untreated, 0.8 mL GL/g-DS, 1.0 mL GL/g-DS and 1.5 mL GL/g-DS pretreated SCB, respectively. **a** Sugars (glucose and xylose) concentration; **b** Ethanol yield (% of the theoretical) and ethanol concentration; **c** Glycerol concentration; and **d** Acetic acid concentration

The solid loading entering the stream of hydrolysis and fermentation affects the conversion efficiency of the pretreated SCB [40, 41]. SSCF of different pretreated SCB at a higher solid loading of 10% (w/v) was carried out under the same conditions as described before, and the results are shown in Fig. 3. The initial concentrations of glucose and xylose were significantly higher than those at the 5% (w/v) solid loading. Compared with that in the untreated SCB, glucose in the pretreated SCB was completely consumed within 17 h. The highest xylose concentration was reached at 17 h, which was longer than the time taken in the SSCF using 5% (w/v) solid loadings. Thereafter, xylose was consumed gradually as the fermentation time increased (Fig. 3a). The metabolic mechanisms that CSC and TSC use to consume glucose and xylose can be expected to be the same as in the previous case (Fig. 2a). It is also possible that in the early stages, the reaction kinetics were limited by the fermentation step due to a high concentration of the sugars, which were found to be correlated with the concentration of the pretreated SCB at the beginning of the SSCF. An increase in the concentration of the pretreated SCB prolonged the time taken to deplete the sugars [34]. The maximum ethanol concentration was 42.53 g/L (0.43 g/g), which was 1.83 times higher than that at the solid loading of 5% (w/v) (Figs. 2b, 3b) using 1.5 mL GL/g-DS in GL–Ethanol pretreatment. Our results are comparable to those obtained with other strains expressing xylose isomerase, such as that developed by Kuyper et al. [42], in which ethanol yields of 0.40–0.43 g/g were achieved during the fermentation of a mixture of 20 g/L glucose and 20 g/L xylose. Our results indicated that the cofermentation of glucose and xylose achieved higher ethanol concentration compared with that obtained by the fermentation of glucose only. One reason for this result was that the coutilization of xylose increased the productivity per unit of SCB added to the fermentation. Alternatively, a low glucose-to-xylose ratio was maintained in the SSCF process, and this facilitated the metabolism of xylose by the TSC yeast due to its affinity for xylose and efficient xylose uptake [7, 12]. However, it is noteworthy that the rate of xylose consumption as well as ethanol production significantly decreased after 24 h, and 4–8 g/L of xylose remained unutilized after 120 h (Fig. 3a). Therefore, the rate of ethanol production slowed down accordingly. In addition, the ethanol yield of 84.99% (of the theoretical maximum) was lower than that at the 5% (w/v) solid loading (Figs. 2b, 3b). It is likely that a high concentration of the lignocellulosic substrate (≥10%) reduced the final ethanol yield because of the increase in viscosity and inhibitor levels in the fermentation system [32].

Glycerol and acetic acid were the primary byproducts of fermentation. The effect of glycerol on ethanol production during fermentation is significant because the generation of glycerol consumes at least 4% of the carbon source available for the fermentation. Glycerol functions to maintain the balance of NAD^+^/NADH in yeast cells, thus playing an important role in starting ethanol fermentation [43]. Glycerol is obtained under osmotic pressure changes and low oxidation–reduction potential. It is also possible that glycerol acts as a glucose analog, resulting in end-product inhibition of cellulase activity [44, 45]. Therefore, it is important to study the production of glycerol in the ethanol fermentation process. At 5% (w/v) solid loading, glycerol concentration during the fermentation increased with the increase in the GL dosage during GL–Ethanol pretreatment (Fig. 2b). The highest glycerol concentration of SCB pretreated with 1.5 mL GL/g-DS was 1.24 g/L. Our findings indicate that GL–Ethanol pretreatment of SCB produces more fermentation byproducts, and as the yeast cells lack acetaldehyde as a hydrogen acceptor, this results in an increase in NADH as the final production concentration increases [37, 45]. Thus, a higher concentration of glycerol is obtained. However, the glycerol concentration in the different pretreated SCB samples decreased with the increase in the total sugar concentration (glucose + xylose) at 10% (w/v) solid loading (Fig. 3a, c). A previous study indicated that enzymatic hydrolysis was slightly inhibited with 0.2% (w/v) glycerol [44]. According to the current results, the glycerol content was below 0.2% (w/v). Besides, glycerol did not irreversibly inhibit cellulase enzymes [44]. Evidently, more studies are needed to find the concentration at which glycerol inhibits cellulase enzyme. Acetic acid concentration showed a similar trend at both 5 and 10% (w/v) solid loading (Figs. 2d, 3d), and untreated SCB exhibited the highest acetic acid concentration during the entire SSCF process. Acetic acid has been shown to cause increased lag times, decreased growth rates, reduced biomass yields, and even cell death in *S. cerevisiae* cultures [46]. Thus, higher acetic acid concentration in the untreated SCB compared to that in the pretreated SCB may have resulted in the lower ethanol yield from untreated SCB in the SSCF process. In contrast, some reports suggested that the exposure of yeast to an environment containing an appropriate amount of acetic acid caused the synthesis of a special substance that protected the yeast strain and promoted yeast fermentation [45]. Previous studies have reported that neither yield nor productivity was affected by the addition of acetate in a range of 2.0–12.0 g/L [47, 48]. It was obvious that the concentration of acetic acid has no effect on the growth of yeast in this paper. The concentration of byproducts depends on the pretreatment methods and the glucose-to-xylose ratio [31]. Therefore, a suitable glucose-to-xylose ratio should facilitate the cofermentation of glucose and xylose in the SSCF of GL–Ethanol-pretreated SCB.

### Comparison of the effects of mixed CSC and TSC strains on the SSF/SSCF process

**Fig. 4:**
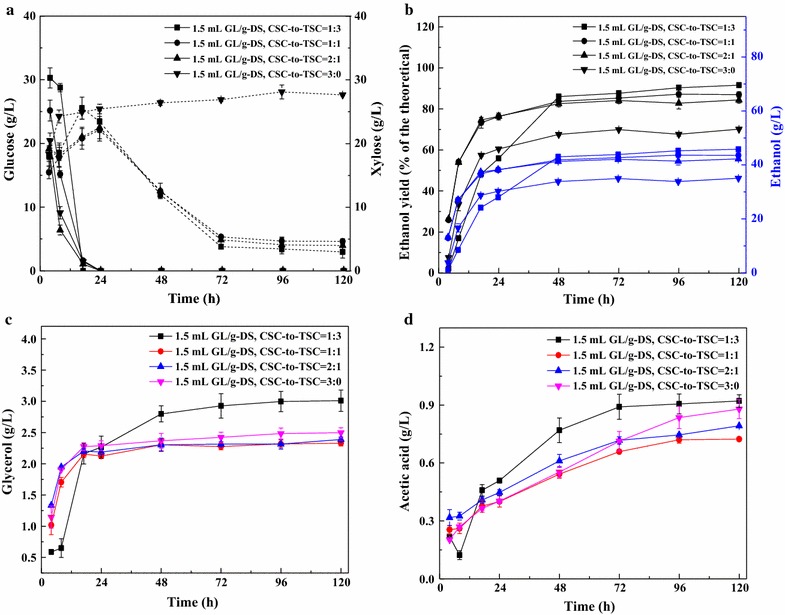
Ethanol fermentation of pretreated sugarcane bagasse (SCB) using 1.5 mL GL/g-DS in GL–Ethanol pretreatment at 10% (w/v) solid loading, and yeast cell mass ratios of 1:3, 1:1, 2:1, and 3:0 (w/w) common *S. cerevisiae* (CSC) to thermophilic *S. cerevisiae* (TSC). Fermentation conditions: 150 rpm, 35 °C, pH 5.5, and 120 h. Square, circular, upright triangle and inverted triangle symbols represent CSC-to-TSC = 1:3, CSC-to-TSC = 1:1, CSC-to-TSC = 2:1 and CSC-to-TSC = 3:0 using 1.5 mL GL/g-DS pretreated SCB, respectively. **a** Sugars (glucose and xylose) concentration; **b** Ethanol yield (% of the theoretical) and ethanol concentration; **c** Glycerol concentration; and **d** Acetic acid concentration

The relationship between the CSC and TSC strains for utilizing glucose and xylose was investigated. Ethanol yield was evaluated from fermentation of pretreated SCB, using 1.5 mL GL/g-DS in GL–Ethanol pretreatment at 10% (w/v) solid loading and CSC-to-TSC yeast cell mass ratios of 1:3, 1:1, 2:1, and 3:0 (w/w) (Fig. 4). Compared to the results obtained using a CSC-to-TSC yeast cell mass ratio of 1:2 (Fig. 3a), we detected higher glucose concentrations in the initial stages of fermentation. Moreover, glucose was almost consumed within 17 h in the SSCF using a CSC-to-TSC yeast cell mass ratio of 1:3, and within 24 h using ratios of 1:1, 2:1, and 3:0, indicating that almost 100% of the glucose was transformed and utilized. For CSC-to-TSC yeast cell mass ratios of 1:3, 1:1, and 2:1, the xylose concentration reached a maximum level (20–25 g/L) at 24 h, and was gradually consumed thereafter. Notably, the ability to utilize xylose increased with an increase in the proportion of the TSC, and 80–90% xylose was consumed in the fermentation (Figs. 2a, 4a). However, for the CSC-to-TSC yeast cell mass ratio of 3:0, the xylose concentration gradually increased to 28.11 g/L in 120 h, probably due to underutilization by the CSC. Our results indicate that during the fermentation process glucose was consumed first, followed by xylose. Furthermore, the TSC strain could probably start metabolizing xylose after glucose concentrations were low and could coferment glucose and xylose effectively [31]. Previous studies have also indicated that hexose catabolism can impede pentose utilization in the SSCF process [49].

The ethanol yields with the CSC-to-TSC yeast cell mass ratios of 1:1 and 2:1 (w/w) were 86.96% of the theoretical maximum (43.52 g/L) and 84.41% (42.24 g/L), respectively (Fig. 4b), and were similar to the yield obtained with the CSC-to-TSC yeast cell mass ratio of 1:2 (Fig. 3b). Importantly, the CSC-to-TSC yeast cell mass ratio of 1:3 showed obvious advantages over the other four CSC-to-TSC yeast cell mass ratios. At 120 h, the highest ethanol yield was 91.57% of the theoretical maximum and the concentration was 45.83 g/L. Consistent with the low utilization of sugar with the CSC-to-TSC yeast cell mass ratio of 3:0, the ethanol yield was only 70.15% of the theoretical maximum, which was markedly lower than that of the other four CSC-to-TSC yeast cell mass ratios. The additional experiment for fermentation with the CSC-to-TSC yeast cell mass ratio of 0:3 showed that the ethanol yield was 80.70% of the theoretical maximum, which was higher than that of using CSC only, but was lower than the yields from mixed CSC and TSC strains. The reason might be that the fermentation using TSC only has a higher conversion rate of xylose, but the glucose inhibitory effect on the TSC is present [50]. Consistent with the previous studies, the SSCF with mixed CSC and TSC strains could significantly improve the utilization rate of hexoses and pentoses, and thus result in higher ethanol concentration. Under similar concentration of mixed sugars, the ethanol concentration of TSC was similarly high (>45 g/L of ethanol) compared with that of other strains reported previously. For example, a strain of xylose isomerase-expressing yeast developed by Kuyper et al. [42] produced only 47 g/L of ethanol from a sugar mixture (100 g/L glucose and 25 g/L xylose). The concentration of the byproducts, glycerol and acetic acid, showed the same growth trend. More importantly, their concentration increased with a greater proportion of the TSC, except for the CSC-to-TSC yeast cell mass ratio of 1:3. Our data are exciting as it indicates a competitive relationship between the two strains and the superior ability of the TSC for glucose and xylose consumption compared to the CSC. This phenomenon was also found in previous cofermentation studies using recombinant *S. cerevisiae* strains [51], and was attributed to the competition between glucose and xylose for hexose transporters [52].

### Comparison of the effect of solid loadings on the SSF/SSCF process

**Fig. 5:**
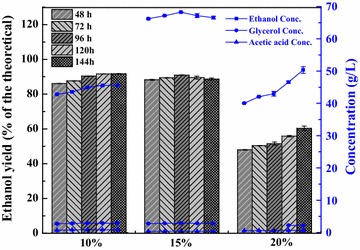
Ethanol yield and concentration of pretreated sugarcane bagasse (SCB) using 1.5 mL GL/g-DS in GL–Ethanol pretreatment at solid loadings of 10, 15, and 20% (w/v) using a yeast cell mass ratio of 1:3 (w/w) common *S. cerevisiae* (CSC) to thermophilic *S. cerevisiae* (TSC). Fermentation conditions: 150 rpm, 35 °C, pH 5.5, and 144 h. Conc., concentration

By using a high solid loading, a high final ethanol concentration has been obtained from cellulosic ethanol. However, a high solid loading of lignocellulosic materials (≥10%) would reduce the final ethanol yield because of the resulting increase in viscosity and levels of inhibitor in the fermentation system [32]. Fermentation of pretreated SCB using 1.5 mL GL/g-DS at 10, 15, and 20% (w/v) solid loadings using the CSC-to-TSC yeast cell mass ratio of 1:3 (w/w) was compared for ethanol production (Fig. 5). To maintain sample mixing uniformity and high activity of yeast cells at high solid loading, the substrate was sampled from 48 to 144 h. Higher pretreated SCB content in the fermenter resulted in lower ethanol yields which are expressed as percent of the theoretical maximum. For example, the ethanol yield with 15% (w/v) solid loading was slightly lower compared to that with 10% (w/v) solid loading, and reached the highest yield of 90.90% (of the theoretical) at 96 h. However, the corresponding ethanol concentration was the highest at 68.24 g/L (Fig. 5), which was higher than that of common cellulosic ethanol fermentation. The comprehensive utilization of GL could be coupled with pulp mills, but the ethanol concentration reduced thereafter. This observed decrease could be due to the reduced metabolic activity of the yeast caused by gradually increasing inhibition from the hydrolysate medium and high ethanol concentration [3]. Alternatively, it is likely that small amounts of ethanol were volatilized over time under the conditions of high solid loading. Remarkably, the ethanol yield with 20% (w/v) solid loading gradually increased after 48 h but reached a maximum of only 60.38% at 144 h, which was significantly lower than the yield obtained with 10 and 15% (w/v) solid loading. To the best of our knowledge, this obvious decrease in yield may have been due to an increased mass transfer resistance and increased inhibitor concentration. Nevertheless, from 48 to 144 h, the concentrations of glycerol and acetic acid with 20% (w/v) solid loading were low (0–3 g/L), which were similar to their levels observed with 10% (w/v) solid loading. Such issues indicated that byproducts concentration might be affected by the mass transfer resistance with respect to solid loading. In addition, acetic acid was not detected until 120 h at 20% (w/v) solid loading (Fig. 5). This implies that the relationship between the solid loading and byproducts also plays a role in the reduction in ethanol metabolic yield. In conclusion, for cost-effective ethanol production and the comprehensive utilization of lignocellulose, SSCF using 1.5 mL/g-DS-pretreated SCB should be an efficient conversion process for ethanol production at 15% (w/v) solid loading with a CSC-to-TSC yeast cell mass ratio of 1:3.

## Conclusions

In this paper, SCB was subjected to GL–Ethanol pretreatment. *S. cerevisiae* strains with different CSC-to-TSC ratios of 1:3, 1:2, 1:1, 2:1, and 3:0 (w/w) were used at 5–20% (w/v) solid loading in the SSCF process. On one hand, this study showed that GL–Ethanol pretreatment is a promising pretreatment method for improving the ethanol production in the SSCF process, due to both better glucan and xylan conversion efficiencies of SCB as well as the higher lignin removal rate. On the other hand, the cofermentation of glucose and xylose with mixed CSC and TSC gave higher ethanol yield than that obtained by the fermentation of glucose alone with CSC. A competitive relationship existed between the two yeast strains, and the glucose and xylose utilization ability of the TSC was better than that of the CSC. In fermentation with 1.5 mL GL/g-DS pretreated-SCB, an increase in the solid loading to a certain extent led to an increase in the ethanol concentration, but at higher solid loadings, the ethanol yield gradually decreased. The highest ethanol concentration reached was close to 70 g/L at 15% (w/v) solid loading with the CSC-to-TSC yeast cell mass ratio of 1:3 (w/w) at 96 h, which was higher than that of common cellulosic ethanol fermentation. The balance between the high solid loadings and the longer fermentation time for economically feasible scenario with respect to large scale process should be compared in the future work.

## Abbreviations

GL: green liquor
GL–Ethanol: green liquor combined with ethanol
SSF/SSCF: simultaneous saccharification and cofermentation
*S. cerevisiae*: *Saccharomyces cerevisiae*
SHF: separate hydrolysis and fermentation
SHCF: separate hydrolysis and cofermentation
SCB: sugarcane bagasse
CSC: common *Saccharomyces cerevisiae*
TSC: thermophilic *Saccharomyces cerevisiae*
CSC to TSC: common *Saccharomyces cerevisiae* to thermophilic *Saccharomyces cerevisiae*
DS: dry substrate
PTFE: polytetrafluoroethylene
NREL: National Renewable Energy Laboratory
HPLC: high-performance liquid chromatography
